# Moral Resilience Reduces Levels of Quiet Quitting, Job Burnout, and Turnover Intention among Nurses: Evidence in the Post COVID-19 Era

**DOI:** 10.3390/nursrep14010020

**Published:** 2024-01-23

**Authors:** Petros Galanis, Ioannis Moisoglou, Aglaia Katsiroumpa, Irene Vraka, Olga Siskou, Olympia Konstantakopoulou, Daphne Kaitelidou

**Affiliations:** 1 Clinical Epidemiology Laboratory, Faculty of Nursing, National and Kapodistrian University of Athens, 11527 Athens, Greece; aglaiakat@nurs.uoa.gr; 2 Department of Nursing, University of Thessaly, 41500 Larissa, Greece; iomoysoglou@uth.gr; 3 Department of Radiology, P. & A. Kyriakou Children’s Hospital, 11527 Athens, Greece; irenevraka@yahoo.gr; 4 Department of Tourism Studies, University of Piraeus, 18534 Piraeus, Greece; olsiskou@unipi.gr; 5 Center for Health Services Management and Evaluation, Faculty of Nursing, National and Kapodistrian University of Athens, 11527 Athens, Greece; olykonstant@nurs.uoa.gr (O.K.); dkaitelid@nurs.uoa.gr (D.K.)

**Keywords:** moral resilience, quiet quitting, job burnout, turnover intention, nurses

## Abstract

The aim of the study was to examine the impact of moral resilience on quiet quitting, job burnout, and turnover intention among nurses. A cross-sectional study was implemented in Greece in November 2023. The revised Rushton Moral Resilience Scale was used to measure moral resilience among nurses, the Quiet Quitting Scale to measure levels of quiet quitting, and the single-item burnout measure to measure job burnout. Moreover, a valid six-point Likert scale was used to measure turnover intention. All multivariable models were adjusted for the following confounders: gender, age, understaffed department, shift work, and work experience. The multivariable analysis identified a negative relationship between moral resilience and quiet quitting, job burnout, and turnover intention. In particular, we found that increased response to moral adversity and increased moral efficacy were associated with decreased detachment score, lack of initiative score, and lack of motivation score. Additionally, personal integrity was associated with reduced detachment score, while relational integrity was associated with reduced detachment score, and lack of initiative score. Moreover, response to moral adversity was associated with reduced job burnout. Also, increased levels of response to moral adversity were associated with lower probability of turnover intention. Moral resilience can be an essential protective factor against high levels of quiet quitting, job burnout, and turnover intention among nurses. This study was not registered.

## 1. Introduction

Nurses, as frontline healthcare professionals, are the most important resource of health organizations, and it is through their contribution that the organizations can achieve their goals. These goals include meeting the health needs of an increasingly ageing population, dealing with complex cases due to the increased number of patients with comorbidities, and reducing errors and adverse events that cause harm to a significant number of hospitalized patients [1,2]. However, health organizations face significant organizational and operational weaknesses, which include understaffing, inadequate resources, poor collaboration, and insufficient leadership support [3]. These weaknesses make nurses’ work challenging, negatively affecting their workplace well-being [4,5] and ultimately leading to failure to achieve organizational goals [6,7]. The COVID-19 pandemic found health organizations struggling with the same organizational problems, further burdening nurses [8], who now had to deal with extremely high workloads as well as significant moral issues when providing care to patients with COVID-19 [9].

One of the longstanding issues of nurses’ well-being, with significant consequences for both nurses and health care organizations, is burnout. It is estimated that burnout internationally affects one in three nurses [10]. Organizational characteristics that have been identified as risk factors for burnout include poor leadership with inadequate support for nurses, understaffing, lack of resources, decreased nurses’ participation in hospital affairs, as well as workload and working longer hours [11,12,13]. The COVID-19 pandemic has placed organizations under extreme pressure, with large numbers of patients requiring emergency and intensive care. Also, during this period, the organizational weaknesses of the healthcare care organizations continued to emerge once again [8]. The combination of extremely difficult working conditions and organizational deficiencies increased the percentage of nurses with burnout to 60% [14]. Nurses who experience high levels of burnout have a compromised quality of life and, although they may still be in the early years of their careers, they are at risk of developing cognitive dysfunction, depression, and impaired sleep [15,16]. On the other hand, insomnia and depression in nurses is associated with the onset of burnout [17]. Consequently, many nurses who experience well-being problems often find themselves trapped in a vicious cycle, where one factor feeds into another, ultimately leading nurses to perpetual mental distress and reduced performance [17,18]. Patients hospitalized in nursing units where nurses experience high levels of burnout report dissatisfaction with care and are at high risk of experiencing an adverse event such as medication errors, infections, and falls [18].

Nurses, in an effort to ensure a healthy working environment, choose to move to another organization or eventually to resign and change career orientation. When nurses’ work environment is characterized by reduced opportunities for promotion, long hours per week with several night shifts, frequent incidents of violence, and high rates of stress and burnout, nurses manifest their turnover intention [19]. When the managements of healthcare organizations recognize the contribution of nurses and work to ensure their well-being, what we call organizational support, then nurses’ work engagement, affective commitment, and satisfaction are increased, and, therefore, a reduction in turnover intention is achieved [19,20,21,22]. Low patient satisfaction, increased likelihood of errors, and high administrative costs are associated with nurses’ turnover [23]. Although turnover intention refers to the likelihood of a nurse leaving an organization in the near future, studies have shown that a significant proportion of nurses ultimately decide to turnover [24]. Therefore, high turnover intention rates are a warning sign that should motivate health service administrations to work towards improving the working environment of nurses.

The COVID-19 pandemic created new working conditions, as all sectors of business were affected by the protection measures and many vacancies were lost. It was, therefore, very difficult for workers, including nurses, to give up their jobs and find a new one. As a consequence, they decided to stay in their jobs but opt for quiet quitting. By choosing quiet quitting, the employee significantly reduces his or her performance by performing completely necessary duties, not expressing new opinions or making suggestions, and avoiding coming in early or staying overtime. She/he basically meets the minimum requirements of the job in order not to be fired [25,26]. In the United States, it is estimated that 50% of business employees have opted for quiet quitting [27]. As it is now difficult for workers to change their working environment, they choose quiet quitting as a form of self-protection from highly demanding working conditions and, at the same time, to achieve a balance between their family commitments and work. Early studies in the health sector show that health professionals also opt for quiet quitting, with nurses showing the highest rates [28]. Nurses who are exhausted are more likely to choose quiet quitting, while job satisfaction plays a mediating role between burnout and quiet quitting [29]. Although quiet quitting is chosen as a reaction to difficult working conditions, it nevertheless seems to be a temporary solution, and ultimately the nurses who choose quiet quitting are those who have higher turnover intention [30].

In their daily practice, nurses are faced with significant challenges that affect them and the quality of care. One of these challenges is moral distress, where nurses are aware of their moral obligation and have decided to act based on their moral principles, but some factors prevent them from implementing their decisions [31]. The factors leading to moral distress can be categorized into three types: clinical situations, internal constraints, and external constraints. The first two types relate mainly to nurses’ lack of knowledge and skills towards the daily situations they have to cope with, while the third relates to organizational and operational dysfunctions [32]. Regardless of its source, moral distress is associated with a negative impact on nurses’ mental health, burnout, turnover intention, and quality of care [33,34,35]. An essential skill for nurses in the management of moral distress is moral resilience, which is defined as “*the capacity of an individual to preserve or restore integrity in response to moral adversity*” [36]. Moral resilience can be cultivated through mindfulness meditation, ethics education, and improved organizational support [37] and can have a protective role in the challenging working conditions of nurses. Through educational interventions aimed at enhancing nurses’ resilience, it is possible to increase work engagement, reduce burnout, and reduce turnover intention [38]. In particular, these programs (mindfulness and meditation) include practices such as the pausing, noticing, and connecting to one’s inner resources and deepest intentions, as well as ethical competence, communication, and resilience training to help nurses better address ethical challenges. In terms of organizational support, this includes the creation of environments that emphasize a culture of ethical practice [37]. The positive outcomes of such educational interventions appear to last for months, providing improvements in nurses on a wide range of moral and mental health issues such as ethical confidence, moral competence, resilience, work engagement, mindfulness, emotional exhaustion, depression, and anger [39]. As moral distress often fosters burnout and nurses’ turnover intention, this creates a mixture of difficult working conditions and burden [40,41]. In addition, burnout can also reduce resilience levels, which in turn affects turnover intention. Nurses are therefore trapped in a vicious circle where each factor can feed into the other, thus perpetuating a distressing situation for nurses [42]. Therefore, the establishment of a supportive work environment through nurses’ education and support team, as well as the implementation of specific strategies by supervisors, is considered fundamental for the promotion and enhancement of nurses’ moral resilience [43,44]. Studies of healthcare professionals in different settings have shown that the higher the moral resilience, the higher the engagement with the healthcare organization and the lower the level of burnout and turnover intention [45,46,47].

To the best of our knowledge, this is the first study that investigated the impact of moral resilience on levels of quiet quitting among nurses. Moreover, we examined the impact of moral resilience on job burnout and turnover intention. In other words, the aim of our study was to investigate the impact of moral resilience on quiet quitting, job burnout, and turnover intention in a sample of nurses after the COVID-19 pandemic.

## 2. Materials and Methods

### 2.1. Study Design

We conducted a cross-sectional study in Greece. Data collection was performed during November 2023. The eligibility criteria for our participants were the following: (a) nurses who have been working in healthcare services for at least two years, (b) those who understood the Greek language, and (c) those who have been working as clinical nurses. Healthcare services included hospitals, health centers, and nursing homes.

We obtained a convenience sample in our study. First, we created an anonymous online version of the study questionnaire using Google forms, and then we disseminated it through Facebook, Instagram, Viber, and WhatsApp. Regarding Facebook, we also posted the questionnaire on nursing groups. Second, we sent the questionnaire to nurses using our e-mail contacts. Finally, we conducted paper-and-pencil interviews with nurses who wanted to participate in our study without using the online questionnaire.

Considering a low effect size (f^2^ = 0.02) of moral resilience on quiet quitting, job burnout, and turnover intention, the number of independent variables (four predictors and five confounders), a confidence level of 99%, and a margin of error of 5%, sample size was estimated at 921 nurses.

### 2.2. Measures

We used the revised Rushton Moral Resilience Scale (RMRS) to measure moral resilience among nurses [48]. The revised RMRS includes 16 items and demonstrates a four-factor structure: responses to moral adversity, personal integrity, relational integrity, and moral efficacy. Each factor includes four items. Score on each factor ranges from 1 to 4. Higher values indicate higher levels of moral resilience. The RMRS-16 has been validated in the Greek language by Katsiroumpa et al. using the forward–backward method to translate the English version of the scale into the Greek language [49]. Authors performed confirmatory factor analysis, and they confirmed the four-factors structure of the scale: response to moral adversity, personal integrity, relational integrity, and moral efficacy. Moreover, they found that Cronbach’s alpha for the factors “responses to moral adversity”, “personal integrity”, “relational integrity”, and “moral efficacy” were 0.652, 0.795, 0.678, and 0.640, respectively. Additionally, the Greek version of the RMRS showed very good concurrent validity as indicated by the statistically significant correlations between the scale and moral distress and job burnout. In our study, Cronbach’s alpha for the factors “responses to moral adversity”, “personal integrity”, “relational integrity”, and “moral efficacy” were 0.665, 0.782, 0.783, and 0.765, respectively.

We used the Quiet Quitting Scale (QQS) to measure levels of quiet quitting among nurses [50]. The QQS has a three-factor structure: detachment (four items), lack of initiative (three items), and lack of motivation (two items). Galanis et al. developed the scale in 2023 using a sample with employees from public and private sector in Greece. They examined several types of validity and reliability for the QQS (i.e., content validity, face validity, construct validity, concurrent validity, test-rest reliability analysis, and internal reliability analysis), and they found that the scale had robust psychometric properties. Authors found that Cronbach’s alpha for the factors “detachment”, “lack of initiative”, and “lack of motivation” were 0.707, 0.706, and 0.747, respectively. Score on each factor ranges from 1 to 5. Higher values indicate higher levels of quiet quitting. We used a suggested cut-off score for the QQS to divide nurses into quiet quitters and non-quiet quitters [51]. In our study, Cronbach’s alpha for the factors “detachment”, “lack of initiative”, and “lack of motivation” were 0.722, 0.714, and 0.747, respectively.

We used the single-item burnout measure to measure job burnout [52]. In particular, we used the valid Greek version of the single-item burnout measure [53]. Authors employed the forward–backward method to translate the tool into Greek. They found statistically significant correlations between the single-item burnout measure and job burnout and general health in a sample of nurses in Greece. Moreover, they found that the single-item burnout measure had high discriminant validity and excellent reliability. The single item burnout measure takes values from 0 (not at all burnt out) to 10 (extremely burnt out).

We used a six-point Likert scale to measure turnover intention among nurses [54]. In that case, we asked nurses, “How often have you seriously considered leaving your current job?” Answers were never (1), rarely (2), sometimes (3), somewhat often (4), quite often (5), and extremely often (6). According to the developers of the scale, values ≤ 3 denote low level of turnover intention, while values ≥ 4 denote high level of turnover intention.

Moreover, we considered gender (females or males), age (continuous variable), understaffed department (no or yes), shift work (no or yes), and work experience (continuous variable) as potential confounders in the relationship between moral resilience and quiet quitting, job burnout, and turnover intention. Nurses who worked evening/nights/weekends were considered as shift workers, while those who worked only mornings from Monday to Friday were considered as non-shift workers.

### 2.3. Ethical Issues

The Ethics Committee of the Faculty of Nursing, National and Kapodistrian University of Athens approved our study protocol (approval number 474, November 2023). We conducted our study on an anonymous and voluntary basis. We obtained informed consent from nurses. Additionally, we conducted our study in accordance with the Declaration of Helsinki [55].

### 2.4. Statistical Analysis

We use numbers and percentages to present categorical variables. Moreover, we use mean, standard deviation, median, minimum value, maximum value, skewness value, and kyrtosis value to present continuous variables. We employed Kolmogorov–Smirnov test and Q-Q plots to assess the distribution of continuous variables. We found that scores on moral resilience, quiet quitting, and job burnout followed normal distribution. Therefore, we used parametric tests such as linear regression analysis to examine relationships between independent (moral resilience) and dependent variables (quiet quitting and job burnout). Moreover, we used linear regression analysis to identify the impact of moral resilience on quiet quitting and job burnout. Since the turnover intention was a dichotomous variable (low versus high level of turnover intention), we used logistic regression analysis to examine the impact of moral resilience on turnover intention. First, we performed univariate regression analysis with quiet quitting, job burnout, and turnover intention as the dependent variables. Then, we constructed multivariable linear and logistic regression models to assess the net impact of moral resilience by eliminating the following confounders: gender, age, understaffed department, shift work, and work experience. In multivariable models, we included simultaneously the four factors for the moral resilience scale (i.e., “responses to moral adversity”, “personal integrity”, “relational integrity”, and “moral efficacy”) and the confounding factors (i.e., gender, age, understaffed department, shift work, and work experience). In case of linear regression analysis, we present coefficient betas, 95% confidence intervals (CI), *p*-values, and R^2^. In case of logistic regression analysis, we present odds ratios, 95% confidence intervals, *p*-values, and R^2^. We considered results as statistically significant when *p* < 0.05. Moreover, when the 95% CIs for odds ratios in the logistic regression models did not include 1, there were statistically significant relationships between variables. Similarly, when the 95% CIs for coefficient betas in the linear regression models did not include 0, there were statistically significant relationships between variables. IBM SPSS 21.0 (IBM Corp. Released 2012. IBM SPSS Statistics for Windows, Version 21.0. Armonk, NY, USA: IBM Corp.) was used for statistical analysis.

## 3. Results

### 3.1. Demographic Characteristics

The study population included 957 nurses (Table 1). Most nurses were females (88.2%) and shift workers (76.3%). Moreover, 78.5% of nurses reported that they work in understaffed departments. The mean age was 36 years, while the mean number of work experience was 10.9 years.

### 3.2. Study Scales

Descriptive statistics for the study scales are shown in Table 2. The mean moral resilience score was 2.87, indicating moderate levels of moral resilience in our sample. We found higher levels of resilience regarding personal integrity (mean = 3.41) and moral efficacy (mean = 3.05) and lower levels of resilience regarding relational integrity (mean = 2.71) and response to moral adversity (mean = 2.29).

The mean quiet quitting score was 2.43. Using the cut-off point for the quiet quitting scale, 71.9% (n = 688) of nurses were considered as quiet quitters, while 28.1% (n = 269) were considered as non-quiet quitters. Levels of lack of motivation (mean = 2.91) were higher than levels of lack of initiative (mean = 2.40) and levels of detachment (mean = 2.20) in our sample.

The mean job burnout score was 7.29, indicating a high level of burnout among nurses. Among our sample, 51.8% (n = 496) reported a high level of turnover intention, with values on turnover intention scale ≥ 4, while 48.2% (n = 461) reported a low level of turnover intention with values on turnover intention scale ≤ 3.

### 3.3. Impact of Moral Resilience on Quiet Quitting, Job Burnout, and Turnover Intention

We found a negative relationship between moral resilience and quiet quitting. In particular, our multivariable linear regression analysis identified that increased response to moral adversity and increased moral efficacy were associated with decreased detachment score, lack of initiative score, and lack of motivation score. Moreover, personal integrity was associated with reduced detachment score, while relational integrity was associated with reduced detachment score, and lack of initiative score. Table 3 shows linear regression models with quiet quitting as the dependent variable.

Additionally, multivariable linear regression analysis revealed a negative relationship between response to moral adversity and job burnout (Table 4). We did not find statistically significant relationships between personal integrity, relational integrity, and moral efficacy and job burnout (Table 4).

Similarly, according to our multivariable logistic regression model, increased levels of response to moral adversity were associated with lower probability of turnover intention. The three other aspects of moral resilience (personal integrity, relational integrity, and moral efficacy) did not have an impact on turnover intention. We present logistic regression analysis with turnover intention as the dependent variable in Table 5.

## 4. Discussion

We conducted a cross-sectional study with a sample of nurses in Greece during the post COVID-19 era. After elimination of confounders, we found a negative relationship between moral resilience and quiet quitting, job burnout, and turnover intention. In other words, our findings showed that moral resilience can reduce levels of quiet quitting, job burnout, and turnover intention among nurses. Additionally, our nurses showed moderate levels of moral resilience, and high levels of quiet quitting, job burnout, and turnover intention.

Quiet quitting is a modern phenomenon that emerged in the COVID-19 era, and its study is still limited in the health sector. The present study is the first to explore the relationship between moral resilience and quiet quitting. As nurses over time work under difficult conditions with limited organizational support, they find that these difficult conditions are worsening and the management of health care organizations are failing to improve them. Nurses have, therefore, decided to take action against this situation; they choose quiet quitting by reducing their efforts, choosing not to let this burden affect them and to achieve a work-life balance. Reducing effort, however, can have consequences for patient care, which is already often plagued by a significant number of adverse events, errors and missed care [56,57]. The effort of the administrations of health care organizations to achieve better outcomes must undoubtedly be based on the effectiveness of the nursing staff, who often work under moral distress situations. Although the relationship between moral resilience and quiet quitting behavior has not yet been investigated, there are studies showing the negative effect of moral resilience on the three subscales of quiet quitting (detachment, lack of initiative, lack of motivation) [45,58,59]. Moral resilience can be cultivated both through training or coach role programs and through the ongoing support of supervisors through specific strategies [37,43,44]. Also, at the organizational level, the interventions which include communication programs, strengthening physician–nurse collaboration, nursing involvement in clinical decision-making and end-of-life issues, social support, using a resiliency bundle, interdisciplinary discussion, and promoting nurses’ ethical and communication skills can enhance the moral resilience of nurses [60].

Although we are in the post COVID-19 era, high levels of nurses’ burnout continue to be recorded. This can be explained by the fact that during the COVID-19 pandemic, nurses worked in extremely difficult situations, and the high levels of burnout continue to affect them [8,61]. In addition to this, situations of moral distress also affected nurses. Limited access to personal protective equipment, fear of transmitting the infection to their family members, caring for patients without family members present, and caring for patients dying without family or clergy present, made up the main moral distress factors that led nurses to withdrawal, burnout, and turnover intention [62,63]. Nurses often find themselves trapped in a vicious cycle where burnout leads to moral distress and then moral distress fuels burnout [62]. Through enhancing nurses’ moral resilience, levels of moral distress and burnout can be reduced [47,62].

The high percentages of nurses who declare their turnover intention should motivate health care administrations, as a significant percentage of nurses who declare turnover intention eventually resign from the profession [24]. Although nurses choose quiet quitting as an alternative to protect them from difficult working conditions, this practice is not enough to keep nurses in their jobs, either. The study showed that nurses who choose quiet quitting are more likely to declare turnover intention [30]. As estimates show that by 2030 there will be 4.5 million nursing staff shortages worldwide, the nurses’ turnover phenomenon will further exacerbate this situation [64]. Organizational support is a protective factor against nurses’ turnover intention [65]. Also, the moral resilience can be an effective tool to halt nurses from leaving the profession, starting even from the youngest nurses, in order to shield them in a timely manner against the difficult working environment they will be facing [47,66].

Undoubtedly, the nursing profession after the COVID-19 pandemic is at a pivotal point. Lack of organizational support and resources, mental health issues, high levels of burnout, quiet quitting, and turnover intentions make up the working environment for nurses, while a shortage of available nurses is expected. All of the above add to an already tired and exhausted nursing workforce, as studies in the pre-COVID-19 era show. Strengthening nursing staff through moral resilience is an important intervention to shield nurses against all of the above factors, without of course overlooking the obligations of the administrations of healthcare organizations to improve nurses’ work environment.

Our study had several limitations. We used a convenience sample of nurses in Greece, and although we achieved the minimum sample size requirements, further studies with representative and random samples would add valuable information. Moreover, studies with nurses in other countries and different clinical settings can expand knowledge. Additionally, we cannot infer a causal relationship between moral resilience and quiet quitting, job burnout, and turnover intention since we performed a cross-sectional study. We used a single item to measure turnover intention, and although this tool is valid, scholars can also use other valid instruments to measure turnover intention. Moreover, we used self-reported questionnaires to measure our variables, and thus information bias is probable in our study. Actual measurement of turnover among nurses can reduce this bias. We eliminated confounding from several demographic and job variables, but several other variables can confound the relationship between moral resilience and quiet quitting, job burnout, and turnover intention, such as managers’ attitudes, personality characteristics, and wage. Moreover, we did not collect data regarding the healthcare services (hospitals, health centers, or nursing homes) that nurses have been working. Thus, we cannot eliminate the confounding effect of different work environments. Our study investigated, for the first time, the impact of moral resilience on quiet quitting in a sample of nurses. Therefore, we should generalize our results with care. Further studies with less bias and different samples should be conducted to improve knowledge.

## 5. Conclusions

Nurses are at the cutting edge of providing quality and safe health care. Although we are in the post COVID-19 era and hospitals have returned to normal operations, nursing staff continue to face significant well-being issues and are ready to leave the profession. Quiet quitting can compromise quality of care; burnout has already been linked to errors, and turnover intention is a precursor to nurses withdrawing, undermining any effort to ensure adequate staffing. Even if the administrations of health care organizations are unable to improve the working environment of nurses, moral resilience can be the intervention that reduces these consequences. Through educational programs, moral resilience can be cultivated, and nurses can promote and maintain a high level of resilience. At the same time, organizational interventions can create a work environment that will reduce moral distress and promote moral resilience [67]. All these can reduce nurses’ likelihood of developing burnout, manifest quiet quitting, and declare turnover intention.

## Figures and Tables

**Table 1:** Demographic characteristics of nurses (N = 957).

Characteristics	N	%
Gender		
Females	844	88.2
Males	113	11.8
Age ^a^	36.0	10.3
Understaffed department		
No	206	21.5
Yes	751	78.5
Shift work		
No	227	23.7
Yes	730	76.3
Years of work experience ^a^	10.9	9.9

^a^ mean, standard deviation.

**Table 2:** Descriptive statistics for the study scales (N = 957).

Scale	Mean	Standard Deviation	Median	Minimum Value	Maximum Value	Skewness	Kyrtosis
Moral resilience	2.87	0.39	2.88	1.31	4.00	0.03	0.26
Response to moral adversity	2.29	0.65	2.25	1.00	4.00	0.19	−0.25
Personal integrity	3.41	0.55	3.50	1.00	4.00	−0.94	0.71
Relational integrity	2.71	0.55	2.75	1.00	4.00	0.27	−0.27
Moral efficacy	3.05	0.49	3.00	1.00	4.00	−0.28	0.21
Quiet quitting	2.43	0.67	2.33	1.00	5.00	0.51	0.38
Detachment	2.20	0.74	2.00	1.00	5.00	0.74	0.81
Lack of initiative	2.40	0.86	2.33	1.00	5.00	0.43	−0.30
Lack of motivation	2.91	0.94	3.00	1.00	5.00	0.32	−0.51
Job burnout	7.29	2.07	8.00	0.00	10.00	−0.91	0.87

**Table 3:** Linear regression models with quiet quitting as the dependent variable (N = 957).

	Detachment Score						Lack of Initiative Score						Lack of Motivation Score					
	Univariate Models			Multivariable Model ^a,b^			Univariate Models			Multivariable Model ^a,c^			Univariate Models			Multivariable Model ^a,d^		
	Coefficient Beta	95% CI for Beta	*p*-Value	Coefficient Beta	95% CI for Beta	*p*-Value	Coefficient Beta	95% CI for Beta	*p*-Value	Coefficient Beta	95% CI for Beta	*p*-Value	Coefficient Beta	95% CI for Beta	*p*-Value	Coefficient Beta	95% CI for Beta	*p*-Value
Response to moral adversity	−0.20	−0.27 to −0.13	<0.001	−0.11	−0.18 to −0.03	0.005	−0.30	−0.38 to −0.22	<0.001	−0.15	−0.23 to −0.06	0.001	−0.37	−0.46 to −0.29	<0.001	−0.31	−0.41 to −0.22	<0.001
Personal integrity	−0.27	−0.35 to −0.18	<0.001	−0.10	−0.20 to −0.002	0.046	−0.42	−0.52 to −0.33	<0.001	−0.04	−0.15 to 0.07	0.44	−0.26	−0.37 to −0.15	<0.001	−0.06	−0.19 to 0.07	0.35
Relational integrity	−0.30	−0.38 to −0.22	<0.001	−0.15	−0.24 to −0.06	0.002	−0.45	−0.55 to −0.36	<0.001	−0.18	−0.28 to −0.08	0.001	−0.26	−0.37 to −0.15	<0.001	−0.01	−0.13 to 0.11	0.89
Moral efficacy	−0.38	−0.47 to −0.29	<0.001	−0.22	−0.34 to −0.11	<0.001	−0.71	−0.82 to −0.61	<0.001	−0.50	−0.63 to −0.37	<0.001	−0.45	−0.57 to −0.34	<0.001	−0.32	−0.47 to −0.17	<0.001

^a^ Multivariable models are adjusted for gender, age, understaffed department, shift work, and work experience. ^b^ R^2^ for the multivariable model = 10.2%, *p*-value for ANOVA < 0.001. ^c^ R^2^ for the multivariable model = 20.8%, *p*-value for ANOVA < 0.001. ^d^ R^2^ for the multivariable model = 12.1%, *p*-value for ANOVA < 0.001. CI: confidence interval.

**Table 4:** Linear regression models with job burnout as the dependent variable (N = 957).

	Univariate Models			Multivariable Model ^a,b^		
	Coefficient Beta	95% CI for Beta	*p*-Value	Coefficient Beta	95% CI for Beta	*p*-Value
Response to moral adversity	−0.60	−0.80 to −0.40	<0.001	−0.65	−0.86 to −0.45	<0.001
Personal integrity	0.38	0.14 to 0.62	0.002	0.22	−0.06 to 0.49	0.12
Relational integrity	0.04	−0.20 to 0.28	0.73	0.10	−0.15 to 0.36	0.43
Moral efficacy	0.24	−0.03 to 0.51	0.08	0.11	−0.21 to 0.43	0.51

^a^ Multivariable model is adjusted for gender, age, understaffed department, shift work, and work experience. ^b^ R^2^ for the multivariable model = 14.4%, *p*-value for ANOVA < 0.001. CI: confidence interval.

**Table 5:** Logistic regression models with turnover intention as the dependent variable (N = 957), (reference category: low level of turnover intention).

	Univariate Models			Multivariable Model ^a,b^		
	Odds Ratio	95% CI for Odds Ratio	*p*-Value	Odds Ratio	95% CI for Odds Ratio	*p*-Value
Response to moral adversity	0.43	0.35 to 0.53	<0.001	0.39	0.31 to 0.50	<0.001
Personal integrity	0.99	0.79 to 1.25	0.94	1.04	0.77 to 1.42	0.79
Relational integrity	0.80	0.64 to 1.01	0.06	1.12	0.84 to 1.50	0.43
Moral efficacy	0.83	0.64 to 1.08	0.17	1.04	0.73 to 1.50	0.82

^a^ Multivariable model is adjusted for gender, age, understaffed department, shift work, and work experience. ^b^ R^2^ for the multivariable model = 16.8%. CI: confidence interval.

## Data Availability

The data presented in this study are available on request from the corresponding author.

## Public Involvement Statement

There was no public involvement in any aspect of this research.

## Guidelines and Standards Statement

This manuscript was drafted against the (STROBE) for a cross-sectional study, descriptive research.
